# The Risk of Atrial Fibrillation and Previous Ischemic Stroke in Cognitive Decline

**DOI:** 10.3390/jcm13144117

**Published:** 2024-07-14

**Authors:** Tunde Pal, Dragos-Florin Baba, Zoltan Preg, Eniko Nemes-Nagy, Kinga-Ilona Nyulas, Marta German-Sallo

**Affiliations:** 1 Department of Internal Medicine V, George Emil Palade University of Medicine Pharmacy, Science, and Technology of Targu Mures, 540142 Targu Mures, Romania; 2 Department of Cell and Molecular Biology, George Emil Palade University of Medicine Pharmacy, Science, and Technology of Targu Mures, 540142 Targu Mures, Romania; 3 Department of Family Medicine, George Emil Palade University of Medicine Pharmacy, Science, and Technology of Targu Mures, 540142 Targu Mures, Romania; zoltan.preg@umfst.ro; 4 Department of Cardiovascular Rehabilitation, County Emergency Clinical Hospital, 540042 Targu Mures, Romania; marta.german-sallo@umfst.ro; 5 Department of Chemistry and Medical Biochemistry, George Emil Palade University of Medicine Pharmacy, Science, and Technology of Targu Mures, 540142 Targu Mures, Romania; eniko.nemes-nagy@umfst.ro; 6 Department of Clinical Laboratory, County Emergency Clinical Hospital, 540042 Targu Mures, Romania; 7 PhD Student-Doctoral School, George Emil Palade University of Medicine Pharmacy, Science, and Technology of Targu Mures, 540142 Targu Mures, Romania; kinga.nyulas@umfst.ro; 8 Department of Internal Medicine III, George Emil Palade University of Medicine Pharmacy, Science, and Technology of Targu Mures, 540142 Targu Mures, Romania

**Keywords:** cognitive test, atrial fibrillation, detection, cognitive decline

## Abstract

**Objectives**: Our study investigated the inverse relationship between cognitive decline (CD) and the presence of documented atrial fibrillation (AFib), ischemic stroke, heart failure, lower extremity peripheral artery disease, and diabetes mellitus. **Methods**: We conducted a retrospective cross-sectional study between December 2016 and November 2019. A total of 469 patients were enrolled who underwent cognitive evaluation with three cognitive tests (Montreal Cognitive Assessment—MOCA, Mini-Mental State Examination—MMSE, and General Practitioner Assessment of Cognition—GPCOG). We used the standard cut-off values, and the optimal thresholds were obtained from the receiver operating characteristic curves. **Results**: The standard cut-off level of the MOCA (<26 points) was associated with the presence of AFib (OR: 1.83, 95% CI: 1.11–3.01) and the optimal cut-off level with <23 points with ischemic stroke (OR: 2.64, 95% CI: 1.47–4.74; *p* = 0.0011). The optimal cut-off value of the MMSE (<28 points) was associated with the presence of ischemic stroke (OR: 3.07, 95% CI: 1.56–6.07; *p* = 0.0012), AFib (OR: 1.65, 95% CI: 1.05–2.60; *p* = 0.0287), and peripheral artery disease (OR: 2.72, 95% CI: 1.38–5.36; *p* = 0.0039). GPCOG < 8 points were associated with ischemic stroke (OR: 2.18, 95% CI: 1.14–4.14; *p* = 0.0176) and heart failure (OR: 1.49, 95% CI: 1.01–2.21; *p* = 0.0430). **Conclusions**: Our research highlighted the broader utility of cognitive assessment. The MOCA and MMSE scores proved to be associated with documented AFib. Higher cognitive test results than the standard threshold for CD of the MMSE, GPCOG, and lower MOCA scores represented risk factors for the presence of previous ischemic stroke.

## 1. Introduction

Mild cognitive impairment is distinct from normal aging and is a transition state between normal cognitive aging and dementia [1]. To have a fast diagnosis of cognitive decline (CD), various tests such as the Montreal Cognitive Assessment (MOCA), the Mini-Mental State Examination (MMSE), and the General Practitioner Assessment of Cognition (GPCOG) have been developed [2]. Previously, cardiovascular diseases (CVDs) were associated with CD, especially atrial fibrillation (AFib), heart failure (HF), and coronary heart disease [1]. In patients with AFib, a higher burden of CD was observed, independent of the occurrence of stroke [1,3]. Furthermore, patients with cognitive decline have higher all-cause mortality rates and CVD deaths as well [4].

AFib is the most common arrhythmia; it could be asymptomatic or discovered in the context of an ischemic stroke, representing the most threatening and disabling complication of AFib [5]. Anticoagulation therapy reduces the risk of ischemic stroke and other thromboembolic events [6]. Several risk factors have been identified that contribute to AFib and to ischemic stroke as well [5,6]. Early detection of AFib is warranted to prevent AFib-related outcomes, such as heart failure, cognitive dysfunction, and impaired quality of life. The most common screening methods for the detection of AFib are a 12-lead electrocardiogram, Holter monitoring, and pulse measurement [6]. Furthermore, due to emerging technological improvements, smartphones and wearable devices have been developed and validated to detect AFib [7]. The current guidelines recommend using the CHA_2_DS_2_-VASc score (congestive heart failure, hypertension, age, diabetes mellitus, prior stroke or transient ischemic attack or thromboembolism, vascular disease, age, sex) to estimate the risk of ischemic stroke in patients with AFib and, at the same time, to indicate anticoagulation therapy to prevent thromboembolic events [6]. 

In light of this, we conducted an observational study to investigate the inverse relationship between cognitive decline assessed by cognitive screening instruments and the presence of documented AFib, ischemic stroke, HF, peripheral artery disease of the lower extremities (PAD), and diabetes mellitus (DM). The secondary objective was to compare the associations between CD test scores and the CHA_2_DS_2_-VASc score and the presence of documented AFib episodes and ischemic stroke. 

## 2. Materials and Methods

### 2.1. Study Design and Population

A retrospective cross-sectional study was carried out in a cardiovascular rehabilitation clinic. Patients hospitalized between December 2016 and November 2019 and having cognitive assessments at admission were enrolled. Screening for cognitive impairment and dementia is a core component of patient assessment admitted to cardiovascular rehabilitation. We included individuals with the following conditions: cardiovascular risk factors, such as arterial hypertension, diabetes mellitus diagnosed during admission or already on antihypertensive or antidiabetic medication; documented AFib based on 12-lead electrocardiogram, Holter monitoring or documented paroxysmal AFib; chronic coronary syndrome including a history of stable angina pectoris, myocardial infarction, surgical or percutaneous coronary intervention for significant coronary lesions; heart failure with established diagnoses in the past or during admission; lower extremity peripheral and carotid artery disease evidenced either by angiography or Duplex ultrasound considering atherosclerotic lesions >50%; history of ischemic stroke. Another inclusion criteria was the cognitive assessment with three cognitive tests (MOCA, MMSE, and GPCOG). The MOCA and MMSE tests are among the most applied and widely validated screening tools in clinical practice and research [8]. The GPCOG, being a brief test administered in primary care, was chosen as a tool for primary care physicians in the case of the applicability of the study’s results. Patients without data on cognitive status (all three tests) and/or documented cardiovascular and cerebrovascular diseases were excluded. It should be mentioned that patients with functional disabilities were not evaluated for cognitive function in the prior testing. Those included severe hearing loss, severe visual impairment or weakness of the dominant arm, which were measured through the patient’s inability to sign the patient consent, and tremor. All patients underwent clinical assessment, blood sampling, a 12-lead electrocardiogram, and echocardiography. Based on these investigations, it was indicated that a Holter examination and peripheral artery ultrasound, including the examination of the lower extremities, extracranial carotid, and vertebral arteries.

Patients gave informed consent to participate in the study before the cognitive assessment. The study was consistent with the Declaration of Helsinki and was approved by the Ethics Committees of the George Emil Palade University of Medicine, Pharmacy, Science, and Technology of Targu Mures and the County Emergency Clinical Hospital of Targu Mures.

During the inclusion period, 536 patients were enrolled in the cognitive evaluation, 65 participants had missing data on a cognitive test, and two more were excluded due to the lack of data on clinical characteristics. Finally, 469 patients were included in the final evaluation. 

### 2.2. Evaluation of Cognitive Function

At the time of admission, we assessed cognitive abilities. Three cognitive batteries were utilized on the same day: MOCA, MMSE, and GPCOG. The assessment was carried out in the afternoon, when participants were not involved in any other investigation, in a quiet room without a third person. The MOCA was developed for the detection of mild cognitive impairment and is a multidomain questionnaire to evaluate global cognition in a paper-and-pencil format. The administration time usually takes 10 min; the obtained scores range from 0 to 30 points, and normal cognition denotes between 26 and 30 points. The education level below or equal to twelve classes requires correction with one additional point to the final score [9]. The MMSE is a well-known and validated screening instrument for detecting dementia. Similar to MOCA, it also tests multiple domains of cognition and takes approximately ten minutes to administer. The maximum score is 30 points, and below 24 points indicates cognitive impairment [10]. Finally, the GPCOG paper-and-pencil test is a shorter instrument for cognitive screening developed for cognitive evaluation in primary care settings. It has two parts, the patient and the informant interview, and takes up to five minutes to administer. The total score for patient assessment ranges between 0 and 9, and we defined the cut-off value as 5 points. The second part of the test is indicated in certain situations (if the patient obtained between 5 and 8 points). However, administering only the patient form has an accuracy of about 90% [11]. Due to the lack of the second part of the test in the majority of the patients (88% of the participants), we considered it necessary to omit the second part in further analyses. We administered the Romanian and Hungarian versions of the tests, as multiple ethnicities are present in the Transylvanian region of Romania. The results were collected in a database, including patient characteristics, cardiovascular risk factors, CVDs, and comorbidities. 

### 2.3. Statistical Analyses

Using MedCalc version 19 for quantitative data, we determined the mean values and standard deviation. We used the Shapiro-Wilks test for normality analyses. We compared the values between the AFib and non-AFib groups using the student’s *t*-test for parametric data and the Mann-Whitney U test for non-parametric data. The associations for categorical values were made using the Chi-square (χ^2^) test. In logistic regression, we determined the associations between standard cut-off levels of MOCA, MMSE, and GPCOG scores and the presence of AFib, ischemic stroke, DM, PAD, and HF. Secondly, we established the cut-off values for all three tests for the identification of previous diagnoses of AFib and ischemic stroke using receiver operating characteristic (ROC) curves and Youden’s index for obtaining the optimal thresholds. Secondary logistic regression was performed for our newly determined levels. Finally, ROC curves were made to compare CD tests with the CHA_2_DS_2_-VASc score in the detection of previous episodes of AFib and ischemic stroke. The significance threshold was set at 0.05.

## 3. Results

### 3.1. Patient Characteristics

For the 469 included patients, approximately a quarter of the participants had AFib (25.2%). The mean age was 67.78 years (SD 9.85), and 52.5% were females, with a mean CHA_2_DS_2_-VASc score of 3.98 points (SD 1.44). Looking at AFib patterns, 40 patients (33.9%) had paroxysmal AFib, 28 (23.7%) were persistent, and 50 patients (42.4%) had been diagnosed with permanent AFib. First, we noticed that the subjects in the AFib group presented a significantly higher mean age (73.08 vs. 66.00; *p* < 0.0001). From the investigated cardiovascular pathologies, HF was present in almost half of the patients (49.3%), and a quarter of the participants had AFib (25.2%). Table 1 shows the clinical characteristics of the study participants divided into two groups: the AFib and non-AFib group. 

### 3.2. Cognitive Assessment

The cognitive evaluation showed a mean MOCA score of 22.82 (SD 4.60) points (cognitive impairment below 24 points), while the mean MMSE was 26.21 (SD 3.33) and the GPCOG was 6.41 (SD 2.49). These were higher than the standard cut-off values (24 points for MMSE and 5 points for GPCOG). Patients with AFib presented significantly lower cognitive mean scores (MOCA: 21.99 vs. 23.10, *p* = 0.0234; MMSE: 25.47 vs. 26.45, *p* = 0.0057; GPCOG: 6.08 vs. 6.52, *p* = 0.0160) compared with the non-AFib group. 

**Figure 1:**
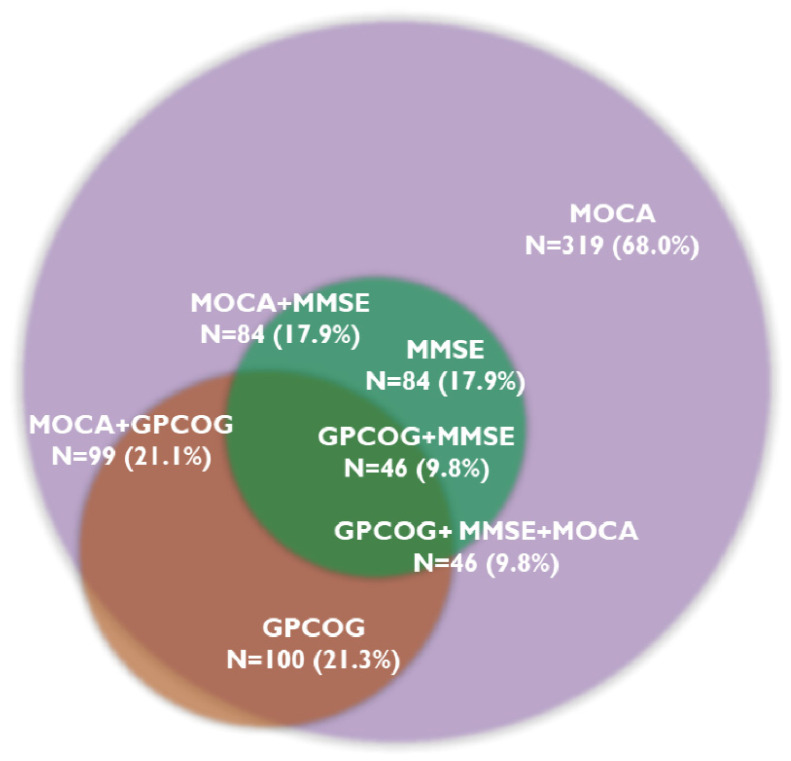
Overlapping prevalence of cognitive decline determined by cognitive tests. Abbreviations: GPCOG, General Practitioner Assessment of Cognition; MMSE, Mini-Mental State Examination; MOCA, Montreal Cognitive Assessment; N, number of patients.

The MOCA test identified cognitive decline in 319 patients (68.0%), and 46 patients (9.8%) showed CD on all three tests. The MMSE test was below the cut-off value in 84 patients (17.9%), while the GPCOG test was in 100 patients (21.3%). Figure 1 presents the prevalence of CD assessed with the three cognitive tests.

### 3.3. Relationship between Cognitive Tests at Standard Cut-Off Levels for CD and the Investigated Diseases

Logistic regression analyses showed a significant association between the MOCA cut-off value for cognitive decline (<26 points) and the presence of documented AFib (OR: 1.83, 95% CI: 1.11–3.01, *p* = 0.0174) and ischemic stroke (OR: 2.09, 95% CI: 1.04–4.22, *p* = 0.0379). At the same time, the MMSE score < 24 points was shown to be a risk factor for the presence of a previous ischemic stroke (OR: 2.43, 95% CI: 1.3–4.53, *p* = 0.0051). The standard cut-off values of the cognitive tests were not associated with DM, PAD, or HF (Table 2).

**Figure 2:**
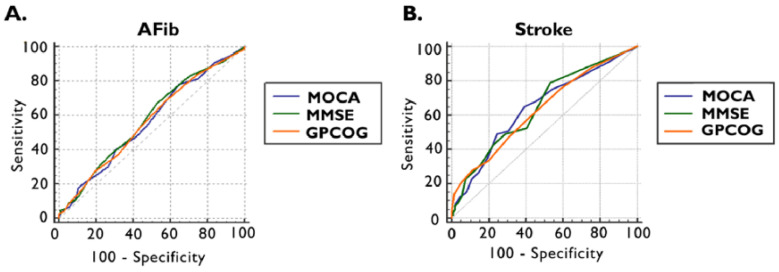
ROC curve of MOCA, MMSE, and GPCOG scores for the presence of documented AFib (**A**) and previous ischemic stroke (**B**). AFib documented atrial fibrillation, GPCOG General Practitioner Assessment of Cognition, MMSE Mini-Mental State Examination, MOCA Montreal Cognitive Assessment, and ROC receiver operating characteristics.

Furthermore, using receiver operating characteristics (ROC) analysis and the maximum Youden index, the optimal cut-off values were established to detect the presence of documented AFib and ischemic stroke by applying cognitive tests (Figure 2 and Table 3). 

**Figure 3:**
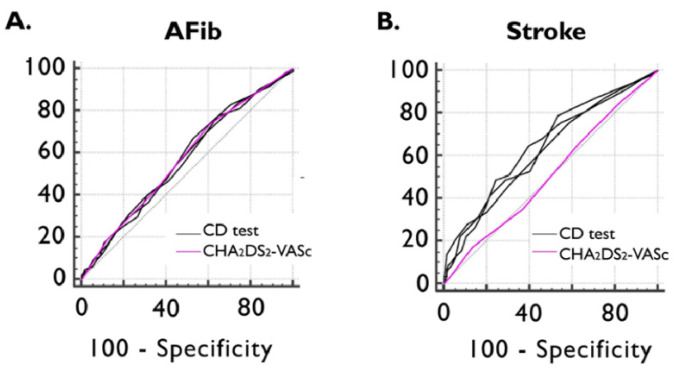
ROC curve of CHA_2_DS_2_-VASc and CD test scores for detecting previously documented AFib (**A**) and ischemic stroke (**B**). AFib documented atrial fibrillation, CD cognitive decline, and ROC receiver operating characteristics.

The ROC curve in the detections of documented AFib for MOCA provided an area under the curve (AUC) of 0.565 (95% CI: 0.519–0.610, *p* = 0.0301) with an optimal cut-off value of <26, which is invariable compared to the standard threshold, with a sensitivity of 78% and a specificity of 35%. The optimal cut-off values in the cases of MMSE and GPCOG were higher than the standards: 28 points for MMSE, respectively, and 8 points for GPCOG. The AUC for MMSE was 0.579 (95% CI: 0.533–0.625, *p* = 0.0074) with a sensitivity of 67% and specificity of 47%, and for GPCOG 0.568 (95% CI: 0.522–0.614, *p* = 0.0215), presenting a sensitivity of 69% and a sensitivity of 42% (Figure 3A and Table 3).

We observed that, by ROC curve analyses, our cognitive tests performed better in detecting ischemic stroke, with a relatively higher AUC for all tests. For MOCA, an AUC of 0.642 (95% CI: 0.597–0.686, *p* = 0.0004) was obtained, with a lower optimal cut-off value < 23 points, leading to a sensitivity of 65% and a specificity of 60%. The MMSE AUC was 0.645 (95% CI: 0.600–0.688, *p* = 0.0002), achieving the cut-off value of <28 points with a sensitivity of 79% and specificity of 47%. In the case of GPCOG, an AUC of 0.631 (95% CI: 0.586–0.675, *p* = 0.0011) was provided, and the optimal cut-off value was <8 points with a sensitivity of 75% and a specificity of 41% (Figure 3B, and Table 3).

Table 2 shows the relationship between the newly defined cut-off values of cognitive tests and the searched pathologies by using logistic regression. All three cognitive tests below the cut-off values were associated with previous ischemic strokes, presenting higher OR than the standard thresholds. There was a statistical association between MOCA < 23 points (OR: 2.64, 95% CI: 1.47–4.74, *p* = 0.0011), MMSE < 18 points (OR: 3.07, 95% CI: 1.56–6.07, *p* = 0.0012), and GPCOG < 8 points (OR: 2.18, 95% CI: 1.14–4.14, *p* = 0.0176) with the presence of previous ischemic stroke. Furthermore, MMSE < 28 points was statistically associated with the presence of documented AFib (OR: 1.65, 95% CI: 1.05–2.60, *p* = 0.0287) and with PAD (OR: 2.72, 95% CI: 1.38–5.36, *p* = 0.0039), respectively, and GPCOG < 8 points with the presence of HF (OR: 1.49, 95% CI: 1.01–2.21, *p* = 0.0430).

ROC curve analyses showed non-inferiority between all three CD tests and the CHA_2_DS_2_-VASc score in detecting documented episodes of AFib. The AUC value of the CHA_2_DS_2_-VASc score in identifying the presence of previous ischemic stroke was 0.514 (95%CI: 0.468–0.561), which was inferior to MOCA (AUC dif: 0.128; *p* = 0.0110), MMSE (AUC dif: 0.130; *p* = 0.0084), and GPCOG (AUC dif: 0.117; *p* = 0.0185) (Figure 3).

## 4. Discussion

**Figure 4:**
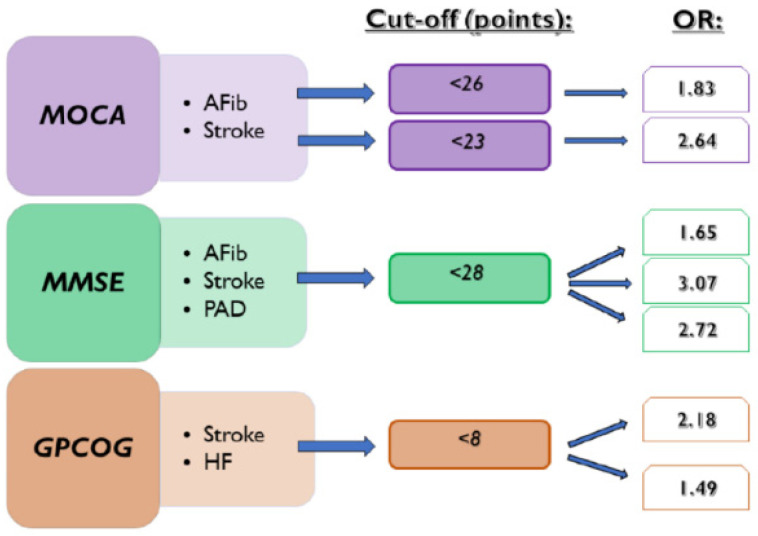
Associations between CD tests and the presence of investigated diseases. AFib documented atrial fibrillation, GPCOG General Practitioner Assessment of Cognition, HF heart failure, MMSE Mini-Mental State Examination, MOCA Montreal Cognitive Assessment, OR odds ratio, and PAD lower extremity peripheral artery disease.

In the present manuscript, we investigate the usefulness of cognitive tests in detecting the presence of various common diseases. The repercussions of AFib occurrence and ischemic stroke on cognitive functions were already demonstrated in previous research [1]. Our results showed that CD test scores can detect the presence of documented episodes of AFib, ischemic stroke, HF, and PAD. We observed that MOCA, MMSE, and GPCOG scores below the cut-off value of 23, 28, and 8, respectively, can be associated with a history of ischemic stroke. The standard cut-off value of MOCA of <26 points used for the assessment of CD also proved to detect the presence of AFib episodes and ischemic stroke. At the same time, MMSE < 28 points was also a risk factor for the presence of documented AFib. Furthermore, we observed a statistically significant association between MMSE < 28 points and GPCOG < 8 points in the presence of PAD, respectively (Figure 4). 

Stroke, CD, and HF represent AFib-related outcomes [6]. Brain magnetic resonance imaging studies showed that AFib was associated with cerebral changes independently of stroke [12]. Participants in the “The Atherosclerosis Risk in Communities Study” (ARIC study) who developed AFib during follow-up had a higher incidence of subclinical cerebral infarctions and worsening ventricular and sulcal grades [12]. Furthermore, in the Swiss Atrial Fibrillation Cohort Study (SWISS-AF), 37% of the patients with AFib had changes in cerebral morphology, and most cerebral infarcts were silent [13]. In this study, large cortical and noncortical infarcts were associated with lower MOCA scores [13]. Our study showed a prevalence of cognitive impairment of 68% assessed by the MOCA test and between 17–21% assessed by the GPCOG and MMSE tests. In a study conducted in European countries among the general population, the prevalence of impairment in different cognitive domains was between 20.75% and 28.02%, with higher values in Hungary (33% and 39%) [14]. Another neighboring country, Bulgaria, reported similar results compared to ours (63% vs. 68%) in patients with comparable clinical characteristics, with higher mean MOCA (23.63 vs. 22.83) and MMSE (27.02 vs. 26.21) scores compared to our study [15]. MOCA is known for its higher sensitivity and lower specificity for assessing CD [10]. A meta-analysis suggested that a lower cut-off value should be used to eliminate false-positive test results in the evaluation of CD [16].

Several methods have been developed for the prediction or screening of AFib [6]. A recent study conducted among primary care patients using the CHARGE-AF clinical score not only successfully assessed the risk of AFib in older patients but also determined a better prediction than the CHA_2_DS_2_-VASc score or age [17]. This score performed better when applied to electronic health records, and even the prediction of AFib-related outcomes, such as stroke and HF, was possible [18]. Nevertheless, it has no clinical implications currently in general practice [6]. The polygenic risk score offered a better prediction for AFib using it together with the CHARGE-AF score and N-terminal pro-brain natriuretic peptide in identifying very high-risk individuals [19]. Results showed promising outcomes based on smartphone and wearable device AFib detection, which further facilitates the diagnosis and management of AFib [20,21]. However, accessibility to devices may be challenging, especially in lower-income countries and in the elderly population. Artificial intelligence earned widespread attention using different variables, such as patient health records and electrocardiograms, to predict AFib [22,23]. Attia and Tiwari reported better discrimination, with an AUC of ≥0.8 for artificial intelligence-based methods compared to our findings [22,23].

Nowadays, the guideline-recommended score to estimate stroke risk in patients with AFib is the CHA_2_DS_2_-VASc score [6]. Research is not limited only to ischemic stroke prediction; it was found that it predicts AFib recurrence after cardioversion and new-onset AFib in patients with ST-elevation myocardial infarction [24,25]. On the other hand, there is evidence that the GARFIELD-AF model (Global Anticoagulant Registry in the FIELD-Atrial Fibrillation) possesses a better prediction of stroke and all-cause death within two years in patients with AFib [26]. The observation that there might be other, more performant risk scores for the prediction of stroke was confirmed in a meta-analysis [27]. It is common knowledge that patients with previous strokes have an increased risk for CD [1]. In patients with CVDs who underwent cognitive assessment, we found an association between cognitive scores and documented ischemic stroke. The standard cut-off values of the MOCA and MMSE tests were detectors of previous strokes. In logistic regression, the optimal cut-off values determined by the Youden index presented a higher risk of the presence of stroke history compared to standard thresholds. In addition, CD scores were superior to the CHA_2_DS_2_-VASc score in identifying the presence of a previous ischemic stroke and non-inferior in detecting documented AFib episodes. The SWISS-AF study investigated the relationship between frailty index (elaborated from 40 variables) and stroke, a secondary outcome in addition to bleeding and death [28]. The authors showed that the frailty index adjusted to age and sex predicted stroke in AFib patients, with a similar AUC to the CHA_2_DS_2_-VASc score [28].

Our findings may supplement the already existing screening methods for detecting AFib and stroke and may have potential benefits besides assessing cognitive function. A major advantage of using a cognitive test-based method for AFib and stroke screening is that patients do not need devices they cannot apply to or afford. Additionally, it is easily accessible and saves resources compared to other methods, such as smartphones, watches, or advanced echocardiography techniques. In a relatively recent study, Kawakami et al. found that left atrial and ventricular speckle tracking techniques have an additive role over risk scores in predicting AFib in patients with a history of cryptogenic stroke [29]. 

In current clinical practice, in certain conditions, such as arterial hypertension, cognitive assessment is part of the evaluation of hypertension-mediated organ damage [30]. Frailty assessment in cardiac rehabilitation is encouraged. Although frailty is present in a higher proportion of elderly patients, it is not exclusively a geriatric condition. A great part of the frailty assessment tools consider the cognitive domain an essential element [31]. Cognitive evaluation is a core component of secondary prevention in cardiovascular rehabilitation, regardless of the main indication for rehabilitation [32]. Regarding the applied questionnaires in our study, two of them, the MMSE and MOCA, are wildely used and recommended cognitive tools in cardiovascular diseases [30,32]. Apart from these situations and conditions, there is a rationale for cognitive evaluation in patients with signs or symptoms of impaired cognition observed by the patient, caregivers, or physicians. Even so, numerous cases remain unrecognized [8]. Based on our results and after substantial improvement of the method in future studies, the application of cognitive batteries, whenever it is indicated, may filter out patients with an increased risk of AFib, ischemic stroke, or other diseases, which may advocate for a mindful and close follow-up and guide further clinical evaluation, which directly impacts patients’ outcomes. 

AFib and HF share risk factors, and AFib patients develop HF in 20–30% of the cases [5,33]. In a Swedish study, lower cognitive test results were associated with increased mortality in hospitalized HF patients and were also associated with rehospitalization [34]. A lower cognitive test score on the GPCOG test was associated with a previous diagnosis of HF in our study. However, this test was not useful for detecting other investigated diseases. 

Integrating our results into the currently available data is difficult. To our knowledge, this study appears to be the first to investigate the detection of documented AFib, HF, and PAD by cognitive batteries. Recently, the recurrence of strokes was assessed with MOCA [35]. We found that even a slightly lower value of the maximum achievable score on cognitive tests could detect the presence of AFib, ischemic stroke, HF, and PAD. The findings of this study bring up facts that require future prospective studies to establish and confirm the predictive role of cognitive tests in certain cardio- and cerebrovascular diseases. Since cognitive screening is recommended in certain patients [8,30,32], based on the test results, screening for AFib, ischemic stroke, HF, and PAD may be relevant, as the presence of these diseases contributes to morbidity and mortality [36]. Certainly, our data must be strengthened first by revising the design of the study for prospective longitudinal research. 

Several limitations of the study are present that require comments. First, the cross-sectional design of this study from a single center emphasizes the need for prospective studies to make clear affirmations about the prediction of AFib or other pathologies using cognitive tests. Second, owing to the single location, our results may not be reliably applicable in different locations, regions, and countries. Third, future longitudinal studies should also investigate the cases of cognitive deterioration, maintenance or eventually improvement, and other prediction factors that contribute to it. Although all included patients were diagnosed by physicians and none of the data were based on self-reported statements, we cannot exclude the possibility of asymptomatic episodes of AFib or undocumented previous AFib and stroke events. In addition, we had no data about the burden of the affected cerebral territory in patients with stroke, and certainly cerebral imagistics could have offered more insights about our associations. Nevertheless, the study sample is less robust than the large populational data, being the first one in Romania, representing start of expanding the research in multi-center studies in order to improve the validity and clinical relevance of the study. Furthermore, the associations between cognitive tests and diseases were significant, though the performance of the applied instruments was not as discriminating compared to other studies mentioned above. Moreover, Romania has a multiethnic population, particularly in the Transylvanian region. Unfortunately, neither ethnicity nor psychosocial risk factors were considered and analyzed as variables. Despite that, future studies should definitely assess these variables in addition to gender and ethnic differences. Last but not least, a successful screening for cognitive function is not equivalent to a diagnosis. A more comprehensive patient evaluation is needed in most cases [8]. 

## 5. Conclusions

In our study, CD was common in patients with cardiovascular diseases. Higher scores on cognitive tests than the standard threshold for CD in the MMSE and GPCOG and lower MOCA scores represented risk factors for the presence of previous ischemic stroke. The standard MOCA cut-off value was shown to detect the presence of documented AFib and ischemic stroke. Our newly determined cut-off value for MMSE was also a risk factor for the presence of documented AFib. Furthermore, lower MMSE and GPCOG scores were associated with the presence of PAD and HF, respectively. Finally, all three CD tests were superior to the CHA_2_DS_2_-VASc score regarding ischemic stroke detection. Future prospective longitudinal studies with larger cohorts are warranted to improve and confirm our results.

## Figures and Tables

**Table 1:** Baseline characteristics. Comparison between the AFib and non-AFib groups.

	General (*n* = 469)	AFib (*n* = 118)	Non-AFib (*n* = 351)	*p*-Value
Mean age, yrs (SD)	67.78 (9.85)	73.08 (7.37)	66.00 (9.95)	<0.0001 *
Gender				
Males, n (%)	224 (47.8)	57 (12.2)	167 (35.6)	0.1521 ***
Females, n (%)	246 (52.5)	61 (13.0)	185 (39.4)	0.1057 ***
Mean education level, classes (SD)	10.73 (2.85)	10.32 (3.09)	10.86 (2.76)	0.0975 **
Mean CHA_2_DS_2_-VASc score, points (SD)	3.98 (1.44)	4.03 (1.41)	3.96 (1.45)	0.6094 **
Cardiovascular risk factors				
Mean BMI, kg/m^2^ (SD)	30.83 (5.96)	30.18 (4.87)	31.05 (6.28)	0.3505 **
Mean AHT, grade (SD)	2.28 (0.54)	2.34 (0.53)	2.27 (0.54)	0.1991 **
Grade III. AHT, n (%)	155 (33.0)	42 (9.0)	113 (24.1)	0.9374 ***
Cognitive evaluation, points (SD)				
Mean MOCA	22.82 (4.60)	21.99 (4.79)	23.10 (4.50)	0.0234 **
Mean MMSE	26.21 (3.33)	25.47 (3.84)	26.45 (3.12)	0.0057 **
Mean GPCOG	6.41 (2.49)	6.08 (3.11)	6.52 (2.24)	0.0160 **
Comorbidities, *n* (%)				
Stroke	57 (12.2)	14 (3.0)	43 (9.2)	0.6953 ***
DM	182 (38.8)	45 (9.6)	137 (29.2)	0.9283 ***
PAD	52 (11.1)	14 (3.0)	38 (8.1)	0.6430 ***
HF	231 (49.3)	60 (12.8)	171 (35.6)	0.5847 ***
CKD	85 (18.1)	20 (4.3)	65 (13.9)	0.9664 ***

* Unpaired *t* test; ** Mann-Whitney test; *** Chi-square (χ^2^) test. AHT arterial hypertension, BMI body mass index, CHA_2_DS_2_-VASc congestive heart failure, hypertension, age, diabetes mellitus, prior stroke or transient ischemic attack or thromboembolism, vascular disease, age, sex, CKD chronic kidney disease, DM diabetes mellitus, GPCOG General Practitioner Assessment of Cognition, HF heart failure, mmHg millimeters of mercury, MMSE Mini-Mental State Examination, MOCA Montreal Cognitive Assessment, n number of patients, PAD lower extremity peripheral artery disease, SD standard deviation, and *yrs* years.

**Table 2:** Association between standard and newly determined cut-off values of CD tests with AFib, ischemic stroke, DM, PAD, and HF.

	Cut-Off Points	AFib OR/95%CI/ *p*-Value	Stroke OR/95%CI/ *p*-Value	DM OR/95%CI/ *p*-Value	PAD OR/95%CI/ *p*-Value	HF OR/95%CI/ *p*-Value
**Standard thresholds for CD tests**						
MOCA	<26	1.83	2.09	1.14	1.48	1.43
		1.11–3.01	1.04–4.22	0.77–1.71	0.75–2.93	0.94–2.15
		0.0174	0.0379	0.4977	0.2500	0.0868
MMSE	<24	1.66	2.43	0.75	1.14	0.68
		0.97–2.84	1.30–4.53	0.45–1.27	0.55–2.39	0.41–1.13
		0.0602	0.0051	0.2849	0.7119	0.1430
GPCOG	<5	0.98	1.26	1.58	- *	0.89
		0.19–5.11	0.14–10.75	0.41–6.09	-	0.21–3.79
		0.9893	0.8317	0.5047	0.9978	0.8816
**Newly determined thresholds for CD tests**						
MOCA	<23	1.25	2.64	0.86	1.56	0.97
		0.81–1.94	1.47–4.74	0.59–1.25	0.86–2.84	0.66–1.43
		0.3059	0.0011	0.4346	0.1392	0.9011
MMSE	<28	1.65	3.07	0.80	2.72	1.46
		1.05–2.60	1.56–6.07	0.54–1.16	1.38–5.36	0.89–2.16
		0.0287	0.0012	0.2452	0.0039	0.0585
GPCOG	<8	1.46	2.18	0.83	1.35	1.49
		0.92–2.30	1.14–4.14	0.57–1.21	0.72–2.53	1.01–2.21
		0.0999	0.0176	0.3417	0.3350	0.0430

* cannot be calculated. AFib documented atrial fibrillation, CD cognitive decline, CI confidence interval, DM diabetes mellitus, GPCOG General Practitioner Assessment of Cognition, HF heart failure, MMSE Mini-Mental State Examination, MOCA Montreal Cognitive Assessment, OR odds ratio, and PAD lower extremity peripheral artery diseases.

**Table 3:** AUC ROC curves of MOCA, MMSE, and GPCOG in the detection of documented AFib and ischemic stroke.

	Cut-Off Points	AUC	Standard Error	95% CI	*p*-Value	Sensitivity	Specificity
**AFib**							
MOCA	<26	0.565	0.029	0.519–0.610	0.0301	78.15	35.43
MMSE	<28	0.579	0.029	0.533–0.625	0.0074	67.23	47.14
GPCOG	<8	0.568	0.029	0.522–0.614	0.0215	68.91	42.29
**Ischemic stroke**							
MOCA	<23	0.642	0.039	0.597–0.686	0.0004	64.91	60.44
MMSE	<28	0.645	0.038	0.600–0.688	0.0002	78.95	46.60
GPCOG	<8	0.631	0.040	0.586–0.675	0.0011	75.44	41.50

AFib documented atrial fibrillation, AUC area under receiver operating characteristics, CI confidence interval, GPCOG General Practitioner Assessment of Cognition, MMSE Mini-Mental State Examination, MOCA Montreal Cognitive Assessment, and ROC receiver operating characteristics.

## Data Availability

The data supporting this study’s findings will be shared upon reasonable request with the corresponding author.
